# Residency training on the frontlines of the COVID-19 pandemic - a qualitative study from Tanzania

**DOI:** 10.11604/pamj.2021.40.28.30919

**Published:** 2021-09-09

**Authors:** Mariam Noorani, Hussein Manji, Elizabeth Mmari, Samina Somji, Nahida Walli, Sherin Kassamali, Shabbir Adamjee, Nancy Matillya, Hanifa Mbithe, Aliasger Nagri, Neelam Ismail

**Affiliations:** 1 The Aga Khan University, Dar es Salaam, Tanzania

**Keywords:** COVID-19, residency training, medical education, Tanzania, Africa

## Abstract

**Introduction:**

the Coronavirus Disease 2019 pandemic has affected residency training globally. The aim of this study was to understand how the pandemic affected teaching and learning in residency programs in low resource settings where residents and faculty were working on the front line treating patients with the disease.

**Methods:**

this qualitative study enrolled residents and faculty from the Aga Khan University in Tanzania who were providing front line care during the pandemic. Purposeful sampling was used and data was collected using focus group discussions and in-depth interviews between August and September 2020. Analysis was done using qualitative content analysis.

**Results:**

twelve residents and six faculty members participated in this study. Two main themes emerged. The first was: “New and unfamiliar teaching and learning experiences.” Residents and faculty had to adapt to changes in the learning environment and the academic program. Residents had increased responsibilities, including providing front line care and working with reduced supervision. The second theme was: “Learning opportunities amidst crisis.” There were opportunities to improve critical care and procedural skills. They also had opportunities to improve non-technical skills like teamwork and communication.

**Conclusion:**

residents and faculty had to adapt to changes in teaching and learning. Residents also had to take up additional responsibilities. Support systems are required to help them adapt to the changes and settle in their new roles. There were opportunities to learn new skills, and training should be restructured to maximize the use of these opportunities.

## Introduction

Coronavirus Disease 2019 (COVID - 19) is caused by the novel coronavirus SARS-CoV-2. The first case was identified in Wuhan city in China, and it then rapidly spread throughout China and then globally, causing a pandemic [1]. The first case in Tanzania was confirmed on the 16^th^ of March 2020 in Arusha in Northern Tanzania as an imported case from Belgium and since then there were reports of community spread with cases being reported in different parts of the country [2]. The effects of COVID - 19 have been numerous and widespread across different domains. It has burdened health care systems around the world and also impacted mental health, caused economic hardships and affected educational systems. Medical doctors enrolled in specialist training programs; also known as residency training; have been affected in various ways. To meet the demand of an increased patient surge from COVID-19, residents from different speciality trainings were moved to work on the front lines and help take care of these patients. They also experienced a reduced number of patient volumes in their speciality areas and a reduced number of elective procedures [3]. This led to decreased clinical exposure, reduced hands-on practice in their respective specialities and a restructuring of residency programs [4-6].

Few studies have been reported from the African continent describing the impact of the pandemic on residency training. In Ethiopia, a study done looking at obstetrics and gynaecology residency training found that there was a significant interruption in didactic and clinical teaching, a severe reduction in gynaecology procedures training and almost a complete stop in research activities [7]. Similar findings were reported from surgical residents in Nigeria [8]. The pandemic also resulted in delays in exams and graduation, with residents feeling inadequately prepared for the exams and not having enough cases to feel ready for fellowship or employment [9]. The impact has also been experienced by faculty who have had to quickly adapt to the drastic change to continue teaching, to minimize disruption to the curriculum and to minimize the spread of the disease [10]. Conversely, however, the COVID-19 pandemic has offered some unique learning opportunities; residents in COVID-19 departments can acquire management skills for the critically ill. Moreover, more free time given by reduced workload in the other wards allowed trainees to have more time to study and update themselves on scientific literature [11]. The impact of the pandemic on residency training globally has been obtained from a program perspective using questionnaires and surveys administered to residents [12, 13]. However, the impact of the pandemic from the individual perspectives and experiences of residents and faculty who have been involved directly in the care of COVID-19 patients has not been well described. Moreover, most of the existing data has come from institutions in high-income countries. In low and middle-income countries, health care delivery systems and medical education programs are quite different. The challenges and experiences of residents and faculty in low resource countries are not known. This study, therefore, aimed to explore the experiences of front line residents and faculty in a middle-income country: Tanzania, to provide insight on how the pandemic impacted their training.

## Methods

**Study design:** we undertook a qualitative study in order to explore detailed and complex insights into participant's lived experiences of teaching and learning during the 1^st^ wave of the pandemic.

**Study area:** this study was conducted at the Aga Khan Hospital, Dar es Salaam (AKHD) which is the teaching site for the Aga Khan University (AKU) Postgraduate Medical Education (PGME) programs. AKHD is a private tertiary hospital that serves mostly the urban population of Dar es Salaam. It is one of the hospitals that offered its services during the COVID-19 pandemic. The Aga Khan Hospital provides emergency, outpatient and inpatient care related to COVID-19. AKU has a global presence in three continents providing training in the fields of health care, education, history and communications. During the study period, the AKU campus in Dar es Salaam offered postgraduate medical training in three specialities: Internal medicine, General surgery and Family medicine. There were a total of 29 residents enrolled in the training programs and over 20 members of faculty. When the COVID-19 pandemic surge began in Dar es Salaam, residents and faculty from all the departments were called upon to work in the emergency, outpatient and inpatient units that were providing care to these patients.

**Study population:** the study participants were residents and faculty of AKU who were involved in the care and management of COVID-19 patients. Faculty and residents were excluded if they were involved as participants in other studies related to COVID-19, as this may have caused the participants to have pre-conceived ideas and suggestions. Residents or faculty who were researchers in the present study were also excluded, as were those who tested positive for COVID-19. The significant mental health impact associated with being positive for the disease was likely to bias study findings.

**Sample size and sampling procedure:** only 12 residents met the inclusion criteria and were available to participate. Many residents had tested positive for COVID-19 and were excluded, while others were on external rotations away from the campus at the time of data collection. All 12 available residents were recruited to participate. Similarly, many faculty members also tested positive for COVID-19 and were therefore excluded, and 6 faculty members were recruited.

**Data collection:** focus group discussions (FGD) were used to collect data from residents, and individual semi-structured interviews were used to collect data from faculty members. FGD was the method of choice for the residents because they shared common experiences during that period and discussing these experiences would bring out rich data. For faculty members, individual interviews were more appropriate since they are all from different specialities and have different experiences. Data was collected between August and September 2020, shortly after the end of the first surge of COVID-19 patients. Three FGD sessions were held with four participants each. The FGDs were moderated by a member of the research team (EM) who had experience with qualitative interviewing. Another member of the research team took the role of note-taker/observer. The FGDs were conducted in a closed room with couches arranged in a circular format to allow direct eye contact and comfortable interaction. English was used as the medium of communication, and the participants were requested to turn off cell phones to minimise interruptions. The FGD proceedings were recorded on the interviewers and note-takers smartphones with the participant´s verbal consent. Each FGD lasted between 50 minutes to 1 hour. At the start of the discussion, all participants were asked to state their year of study, their department and the role they played in the pandemic. No names were mentioned to maintain confidentiality. A FGD topic guide was used to lead the discussion, and it had broad open-ended questions. Examples were: “How has the pandemic affected you as residents in your training?”, “Can you describe the specific areas of training that were affected?” Probing questions were further used to obtain rich information. A final open-ended question at the end of the interview was: “Is there anything you would like to add that you think is important regarding residency training during this time of COVID-19?”

Individual interviews were conducted in the offices of the faculty members. A member of the research team (NI) who has experience with qualitative interviewing trained 3 other research team members on how to conduct the interviews. Each interview had a main interviewer and a note-taker. An interview guide was used, with an opening question on the role the faculty member had played during the pandemic. Subsequent questions included: “How has the pandemic affected you as a faculty in your training/teaching?” A concluding question was: “Is there anything you would like to add that you think is important regarding residency training during this time of COVID-19?” Each interview lasted between 20 to 30 minutes. Data collection was done as an iterative process with analysis. Emerging issues and further data collection needs were identified and incorporated into subsequent FGDs and interviews. For example, in the initial FGD, online learning emerged as a concept towards the end of the discussion. This was then incorporated as a specific question for the next FGD: “what was your experience of online learning?”

**Data management:** the audio recorded files were transcribed verbatim by various members of the research team. After transcription, the audio files and the transcripts were all transferred into the lead researcher (MN)´s computer for storage. Individual researchers deleted files from their smartphones.

**Data analysis:** it was carried out using qualitative content analysis [14]. In content analysis, the researcher interprets meaning from the content of text data, which is obtained from experiences and events. This method was used to describe and create meanings of the impact on training and teaching during the COVID-19 pandemic.

Initial analysis for each transcribed interview/FGD was performed by 2 researchers independently. Analysis was carried out by initial reading of the transcribed text several times to gain an insight into the meanings conveyed. Meaning units were identified from the data; these were then condensed and labelled with a code. Both researchers then met to agree on final codes for each transcript. The codes from each interview/FGD were sent to the lead researcher (MN). MN, HM and NI then grouped the codes into sub categories and categories at the manifest level and finally grouped into themes to bring out the latent content. An example of this is shown in Table 1. All team members agreed on final categories and chosen quotations.

**Table 1:** examples of meaning units, codes and sub-categories

Meaning unit	Condensed meaning unit	Code	Sub- category
We were not getting patients who require surgery that much because people were fearful to come to the hospital for surgeries, electives and all so we did not get that much exposure.	Patients feared to come to hospital so lack of surgical skills exposure	Reduced number of elective cases	Decreased volume and case mix of non-COVID cases
And also, I have participated in multiple CPR´s, so this has also added on to my...to my CPR skills which was also very nice.	Participated in CPR and improved skills	Improved resuscitation skills	Critical care training

**Ethical considerations:** ethics approval for this study was obtained from the Ethics Review Committee (ERC) of the Aga Khan University (AKU) which is mandated by the National Institute of Medical Research (NIMR) Reference: AKU/2019/075/fb. Data collection was done after participants had read and signed an informed consent. The participants were identified by using code numbers to protect their identity, such as R1 for resident 1, and F1 for faculty 1. All identifying information was deleted from the transcripts.

## Results

Twelve residents and 6 faculty members participated in the study. There were 4 residents from each of the departments (internal medicine, general surgery and family medicine) and there were 2 faculty members from each department. Two major themes emerged upon analysis of the data: 1) New and unfamiliar teaching and learning experiences. 2) Learning opportunities amidst crisis. These themes were built upon 5 main categories and many sub-categories, as summarized in Table 2.

**Table 2:** themes, categories and sub-categories describing the impact of COVID-19 on residency training

Themes	Categories	Sub-categories
New and unfamiliar teaching and learning experiences.	Changes in the clinical learning environment	Decreased volume and case mix of non-COVID cases increased COVID work load changes in patient-doctor interaction
	Changes in the academic program	Learning objectives affected formal teaching interrupted deadlines and assessments missed experiences and challenges of online learning
	Increased resident responsibilities	Working in new environments role as a frontline worker reduced supervision from faculty
Learning opportunities amidst crisis.	Improved non-technical skills	Teamwork built resilience administrative and communication skills adaptation
	Improved clinical skills	Critical care training procedural skills

### Category 1: changes in the clinical learning environment

The clinical learning environment was completely different as compared to what the residents and faculty members were accustomed to. The major changes in the environment were due to: reduced volume and case mix of non-COVID patients, increased COVID related work load and changes in the doctor-patient interaction. Due to COVID - 19, there was fear in the community about coming to the hospital and getting infected with the virus and as a result there were reduced volumes of non-COVID patients and hence decreased case mix for learning purposes for the residents. Surgical residents were affected the most.

“*…we were not getting patients who require surgery that much because people were…were fearful to come to the hospital for surgeries, electives and all so we did not get that much exposure*” **R3 FGD 2**

Residents were also overwhelmed with work due to the high number of COVID-19 patients which stretched their working hours and contributed to exhaustion. They also had to balance between didactic learning and managing clinical responsibilities.

“*...we had too many cases, it's difficult to study. I mean one day after post call it's difficult to go back and read because you are exhausted but also because of the burden of the patients…*” **R4 FGD 3**

There were also significant changes in the doctor-patient interactions because of fear of contracting COVID-19. Physical examinations were limited and the interaction time between the patient and doctor shortened to reduce the risk of exposure and contracting COVID-19.

“*We were not examining because we were reducing contact, especially with those respiratory related physical exams, ENT exams… At one point we were not completely examining patients we just treat them symptomatically and would just almost like give everything*.” **R2 FGD3**

### Category 2: changes in the academic program

There was a complete disruption of the academic program that had been planned for the year. The learning objectives that had been set were affected, formal teaching was interrupted, deadlines and assessments were missed and everyone had to transition to online learning.

“*…all my academic purpose at that point in time was put to halt...change in timetable, a change in environmental and the pandemic itself made us not to concentrate on our initial objectives...*.” **R3 FGD 1**

Formal teaching was put on hold, and this was emphasized by both residents as well as faculty. The increased workload and change in priorities during the pandemic caused residents to be overwhelmed and thus not having time to prepare for sessions. Also, the need for social distancing was important and so formal academic sessions could not be held.

“*We had to stop everything… and just attend COVID patients... We were all front liners. We were all just attending COVID patients, we had to stop everything. So, there were no journal clubs, there were no case presentations, and most of the residents were assigned to do COVID rounds, to be on call, and at the COVID ward*.” F2

Deadlines were missed, especially for resident dissertations. Assessments that were scheduled were cancelled and formative assessments that are done using workplace-based assessment tools were not filled out and residents were no longer monitoring their individual progress.

“*...it was so busy at one point that all of us were focusing in the service, and you cannot ask the rounding consultant to assess Mini Cex*” R4 FGD 1

To mitigate the changes in the academic program, attempts were made to use online learning platforms to catch up on sessions. Residents and faculty all had to transition to online learning and several challenges were encountered, including finding innovative ways to engage the learners.

“*The challenges that we are facing with these interventions is how do you account for people participation? Are there ways you can say when someone is online is also actively participating, or they have just logged in*” F5

Residents were working different shifts, and so their work schedules affected online learning. Not all residents would be available at the same time, and for those that were, they would be overworked and unprepared for the session.

“*We couldn´t do it because the time we were having our zoom sessions others would be on shift so we had to stop...So during the pandemic, the online session failed because you are either on shift or finished your shift and are exhausted to join the session*.” -**R3 FGD 1**

### Category 3: increased resident responsibilities

The need for an increased work force to manage the large patient volumes resulted in residents having to take up more responsibilities. They had to step up and manage patients as frontline workers, and they also had to manage patients with reduced supervision from their faculty.

“*…after the outbreak, we shifted tasks. We started working in the COVID wards where we worked as front liners. At the same time, we were seeing outpatients which included COVID-19 patients. So we managed both inpatient and outpatient*” **R1 FGD 1**

Most of the residents expressed that they received less supervision from their program supervisors and the specialists. Different reasons contributed to this; the supervisors were also involved in COVID-19 duties while some of the faculty contracted the disease and were not at work.

“*…so you know most of the time residents are supervised…but that particular point in time the supervision was more of an indirect rather than a direct supervision*.” **R2 FGD 2**

### Category 4: improved non-technical skills

The pandemic offered the residents and faculty a unique opportunity to strengthen non-technical skills. They were able to establish teamwork, build resilience and improve their administrative and communication skills. They also had to quickly adapt to new environments, new treatments and new roles.

“*Well I had a new team during the pandemic we were in a group which was named A&E warriors, it was a good experience working with the new team. …I had a new team so I had to start getting used to them, their team work and that itself was an experience…*” **R1 FGD 1**

Due to the increased volumes of COVID-19 patients and those that needed critical care, the residents learnt how to multitask, prioritise and developed resilience. They worked long hours and managed complex clinical situations, including patients with multiple co-morbidities.

“*I learnt how to work in stressful environment. During COVID, it was very stressful with the number of patients. How to keep the priorities for example, you have 8 patients and all of them have to go to the ward so that the emergency can be empty to accept other patients so to plan your priorities.*.” **R4 FGD 3**

One of the responsibilities of the residents was to reveal bad news to the families and relatives of the deceased. In addition, they also had to communicate to patients and families about their COVID test results.

“*..as you know the outcomes were not that good…so once you find there´s a positive…COVID results you will be forced like to practice how to break bad news and in terms where there was death…so you are forced like to break the bad news…*” **R2 FGD 2**

### Category 5: improved clinical skills

The pandemic had a positive impact on the residents´ critical care and procedural skills.

“*…I was in the critical care unit, so I have learnt every technique to improve oxygenation of a patient that I wouldn't have without pandemic…*” **R2 FGD 1**

The residents had shifts in the wards and in the emergency department, where they got opportunities to triage patients and resuscitate those who required emergent care. They acknowledged that this experience improved their resuscitation skills. They also had more opportunities to carry out emergency procedures.

“*…it opened up a window for some of the procedures like if you were interested to intubate a patient or put a central line there was a big chance of doing the procedure because we had very sick patients who required urgent care*.” **R2 FGD 1**

## Discussion

Residency training programs form the backbone of health care service delivery in many health systems. Residents are the key workforce providing clinical care to patients under the supervision of their faculty members. However, in addition to providing clinical care, residents have to meet the academic requirements of their training programs, which may include research work, assessments and achieving required competencies. Our study described how the residents had to deal with increased work load due to the pandemic and their role as front liners in new environments. This situation has been faced by residents in training programs globally where they have had to handle the workload of COVID-19 patients irrespective of their speciality area of training [11, 15]. The surgical residents highlighted that they had reduced case volumes and case mix of surgical cases. This is similar to what has been found globally in surgical residency training programs and resulted in restructuring of many training programs [4, 6, 8]. However, in the case of our programs, although the classroom sessions were restructured to be done virtually, many residents were unavailable to attend because they were on the frontline providing care. The high patient volume in the COVID units resulted in lack of direct supervision from faculty. Residents were practising independently with some indirect supervision. Although this can build confidence in decision-making, it also has the potential to compromise patient safety. The Accreditation Council of Graduate Medical Education (ACGME) reiterated that residents who are managing COVID-19 cases should have adequate supervision by faculty who are trained in such protocols [16].

The change to virtual learning brought with it a new set of challenges for both residents and faculty members who had to navigate through this new experience. There has been concern raised globally that this kind of emergency remote teaching may not be as effective as well-planned online learning sessions [10]. Assessment and research deadlines were missed by the residents during the pandemic surge. Globally, high stakes exams were cancelled or postponed, resulting in delays in graduation and employment [5, 17]. Lack of adequate assessments also raises concern about whether graduates can meet the required competencies for independent practice. Examination boards have recognized that the traditional time-based or volume-based competency measures may not be applicable during a pandemic, and guidelines have been issued to help training programs make competency related decisions [18]. Despite the myriad challenges brought by the pandemic, residents and faculty were able to identify learning opportunities that were created. This type of learning can best be understood using Mezirow´s transformative learning theory [19]. This theory describes how learning is achieved when faced with crisis situations, similar to what residents faced with COVID-19. The key to learning in such situations is a process of critical reflection, which then results in a change in perspective and learning through transformation.

The non-technical skills that were learnt included teamwork, building resilience and leadership. A perspective from Singapore has described how pandemics can improve non-technical skills in trainees such as leadership, teamwork, resource management and adaptability [20]. Apart from the non-technical skills, the residents also acquired critical care skills which are a valuable additional skill set. It will be important to continue to reinforce these skills to ensure they are not lost since skills fade within 6 to 18 months if not practised. [21]. Simulation is one way in which these skills can be reinforced periodically.

### Limitations

Due to the limited number of available participants, thematic saturation could not be ascertained. However, from the analysis of the 3 FGD and 6 interview transcripts, recurring concepts were noted with little variation and hence it is likely that the most relevant data has been obtained from the existing participants.

### Trustworthiness

The credibility of the study was ensured by the use of triangulation. Investigator triangulation was done by having two researchers analyse each transcript and jointly agreed on the codes. Final categorization and theme generation was done jointly by 3 members of the research team with experience in qualitative research analysis. Data triangulation was done by collecting data from 2 different sources - faculty and residents. Transferability was ensured by having participants from various departments and sufficiently describing in detail the study site and study procedures. Dependability and confirmability have been ensured by describing clearly the whole process of the study. The whole research team was trained on the use of qualitative content analysis and strictly followed the laid down procedures. An audit trail has been maintained by the lead researcher to ensure that all analysis can be traced back to the raw data.

## Conclusion

Educational leaders must be cognizant of the experiences of residents and faculty on the front line. This will help them reorganize training and will ensure minimal disruption of academic activities as the pandemic continues. It will also help the residents to meet their learning objectives and deadlines. It is equally important to provide support systems to help them successfully navigate these new experiences. Finding ways to ensure adequate supervision despite large patient volumes will ensure patient safety. The pandemic provided learning opportunities for residents. To maximize the learning, these skills can be included in their learning outcomes and clear teaching and assessment strategies defined for assessing the skills. Continuous reinforcement should also be ensured to prevent loss. Helping residents and faculty to engage in critical reflection of their teaching and learning during this time can also improve learning through transformation.

### What is known about this topic

- The COVID-19 pandemic has affected postgraduate medical training globally;
- Residents in training have had reduced case mix and volume for training.

### What this study adds

- The COVID-19 pandemic has had positive impacts on learning that should be recognized and supported;
- Residents have to adapt to changes in the clinical learning environment and the increased responsibilities that have come with the pandemic.
